# Comparative Efficacy of Ublitixumab Versus Natalizumab in the Treatment of Relapsing and Remitting Multiple Sclerosis

**DOI:** 10.7759/cureus.95422

**Published:** 2025-10-26

**Authors:** Sai V Chitturi, Jonnalagadda Amith Priyansu, Akhil Sadhu, Hemanthi Ramisetty, Manoj R Pallapothu, Deepiikha Kogantii

**Affiliations:** 1 Family Medicine, Pontiac General Hospital, Pontiac, USA; 2 Family Medicine, Faculty of Medicine, St. Martinus University, Willemstad, CUW; 3 Medicine, Guntur Medical College, Guntur, IND; 4 General Medicine, All India Institute of Medical Sciences, Mangalagiri, Mangalagiri, IND; 5 Neurology, Bridgetown International University School of Medicine, Bridgetown, BRB; 6 Research, Faculty of Medicine, St. Martinus University, Willemstad, CUW; 7 Medicine, Pontiac General Hospital, Pontiac, USA; 8 Family Medicine, Apollo Institute of Medical Sciences and Research, Chittoor, IND

**Keywords:** monoclonal antibodies (mabs), natalizumab, relapsing and remitting, s: multiple sclerosis, ublitixumab

## Abstract

Relapsing-remitting multiple sclerosis (RRMS) is a chronic autoimmune disorder characterized by immune-mediated demyelination and neurodegeneration, leading to progressive neurological impairment. Disease-modifying therapies (DMTs) play a crucial role in managing RRMS by reducing relapse frequency and slowing disability progression. Among these, monoclonal antibodies such as ublituximab and natalizumab have emerged as key therapeutic options with distinct mechanisms of action and safety profiles. This narrative review aims to compare the efficacy, safety, and clinical impact of ublituximab and natalizumab in the treatment of RRMS, providing insights into their role in individualized treatment strategies.

## Introduction and background

Relapsing-remitting multiple sclerosis (RRMS) damages the demyelization of the spinal cord and nerve fibers of the brain. Unpredictable changes in the motor, sensory, and cognitive systems, along with related mental and physical comorbidities, are brought on by this neurological process [1]. Women are three times more likely to develop MS than men, and it commonly affects those around age 30. Repeated damage to the myelin sheaths and other parts of the nerves can lead to serious disability. It is distinguished by intervals of neurological malfunction that are followed by recovery. Cell-mediated demyelination, remyelination, microglial activation, and chronic neurodegeneration are among the pathogenic processes that take place in MS. The clinical history, which is marked by neurological dysfunctional episodes that are followed by recovery, attacks that leave lasting deficits, and progression that results in permanent physical and cognitive disability, is influenced by the sequential participation of these systems. MS is one of the most common causes of neurological disability in young people, with an annual incidence that ranges from two to 10 cases per 100,000 persons and a north-south gradient, with a lower prevalence near the equator [2].

Monoclonal antibodies, such as natalizumab and ublituximab, have been created to target particular immune pathways and aid in the efficient management of RRMS. This review compares these two treatments based on efficacy, safety, and clinical outcomes. These monoclonal antibodies have been created to target particular immune pathways and aid in the efficient management of RRMS. Ublituximab and natalizumab stand out for their distinct mechanisms of action and established efficacy. This review aims to provide a direct comparison of ublituximab and natalizumab in the management of RRMS, considering their clinical efficacy, safety, and key differences to inform individualized treatment decisions.

Understanding the different types of MS is essential for tailoring treatment approaches and determining the most effective therapy for individual patients.

Types of multiple sclerosis

Multiple sclerosis (MS) is categorized into four primary forms, each with distinct clinical characteristics:

Relapsing-remitting MS (RRMS): The most frequently diagnosed form of MS, RRMS is marked by clearly defined attacks of new or worsening neurological symptoms (relapses), followed by partial or complete recovery periods (remissions). In between relapses, there is typically no disease progression. Majority of individuals diagnosed with MS initially present with this form.

Primary progressive MS (PPMS): Unlike RRMS, PPMS is characterized by a continuous decline in neurological function from the onset of symptoms, without distinct relapses or remissions. While some patients experience temporary stabilization, most progressively worsen over time. PPMS accounts for around minority of MS cases and often leads to significant disability earlier in the disease course.

Secondary progressive MS (SPMS): Many individuals diagnosed with RRMS eventually transition into SPMS, where relapses become less frequent, and a steady decline in neurological function occurs. While SPMS may still include occasional relapses, disease progression becomes the predominant feature, often leading to cumulative disability.

Progressive-relapsing MS (PRMS): This is a rare subtype of MS that presents with a continuous progression of symptoms from the onset, but with occasional acute relapses. Unlike RRMS, recovery after these relapses is often incomplete, and disability accumulates more rapidly.

## Review

Selection criteria

This narrative review includes peer-reviewed studies and clinical trials published between 2000 and 2024, focusing on the efficacy and safety of ublituximab and natalizumab in treating RRMS. Articles were selected through PubMed, Google Scholar, and NeurologyLive using keywords such as 'ublituximab', 'natalizumab', and 'multiple sclerosis'. Preference was given to large randomized trials and reviews relevant to clinical practice.

Background and pathophysiology

Brainstem and spinal cord syndromes, optic neuritis, and rarer symptoms such as cortical presentations like dominant parietal lobe syndrome are the most prevalent clinical manifestations of multiple sclerosis. Depending on where the brain is involved, clinical signs can vary. An evaluation of the disease phenotypes, including the assessment of disease activity based on imaging findings, clinical relapses, and disease progression, has been conducted by the International Advisory Committee on Clinical Trials of Multiple Sclerosis. Approximately 85% of individuals experience a relapsing-remitting (RRMS) course, which is marked by a series of remissions and deteriorations [3]. B-cell-depleting monoclonal antibodies (mAbs) have demonstrated a substantial correlation with the pathophysiology of MS. Throughout the lifecycle of both naïve and memory B-cells, these monoclonal antibodies target CD20, a cell surface molecule expressed on pre-B and mature B lymphocytes. Fc-gamma receptor (FcγR)-mediated phagocytosis, complement activation, and direct cell killing can all be induced by anti-CD20 mAbs via immune effectors such as macrophages and natural killer (NK) cells [4].

Pathophysiology of multiple sclerosis

The pathogenesis of MS is primarily driven by aberrant immune responses. Autoreactive CD4+ T cells, particularly Th1 and Th17 subsets, are central to initiating the inflammatory cascade. These cells recognize central nervous system (CNS) antigens and, upon activation, cross the blood-brain barrier (BBB), leading to the recruitment of additional immune cells and subsequent demyelination [5].

Interferon gamma and other proinflammatory cytokines are secreted by Th1 cells, while IL-4 and IL-13 are secreted by Th2 cells. The CD4 T cell subset Th17 produces IL-17, IL-21, IL-22, and IL-26. In MS, Th17 cells, like Th1 cells, encourage inflammation. Both acute and chronic plaques of MS contain IL-17 receptors. Studies on animals lacking IL-17 also reveal decreased clinical severity [6].

A different subset of CD4 T cells implicated in the pathophysiology of MS are regulatory T cells (T reg). T regulatory cells have the responsibility of controlling Th1, Th2, and Th17 effector cells. Controls and MS patients have the same amount of T reg cells, but MS patients' T reg function is diminished [7].

B cells also play a pivotal role in MS pathophysiology. They act as antigen-presenting cells, produce autoantibodies, and secrete pro-inflammatory cytokines like interleukin 6 (IL-6) and tumor necrosis factor-alpha (TNF-α). The presence of oligoclonal bands in the cerebrospinal fluid (CSF) of MS patients underscores the involvement of B lymphocytes in the disease process.

Blood-brain barrier disruption

The integrity of the blood-brain barrier (BBB) is compromised in MS, facilitating the entry of immune cells into the CNS. Matrix metalloproteinases (MMPs), produced by activated T cells, degrade the extracellular matrix components of the BBB, enhancing its permeability. This breach allows for the infiltration of autoreactive lymphocytes and monocytes, perpetuating inflammation and tissue damage [8].

Demyelination and axonal damage

Once within the CNS, immune cells target myelin sheaths, leading to demyelination. Macrophages and microglia phagocytose myelin debris, while cytotoxic CD8+ T cells directly damage oligodendrocytes and axons. The resultant axonal injury disrupts neural conductivity, manifesting clinically as neurological deficits. Chronic lesions exhibit gliosis and axonal loss, contributing to the progressive nature of MS. Mitochondrial dysfunction and oxidative stress further exacerbate neurodegeneration, independent of active inflammation.

Mechanism of action of ublitixumab

**Figure 1:**
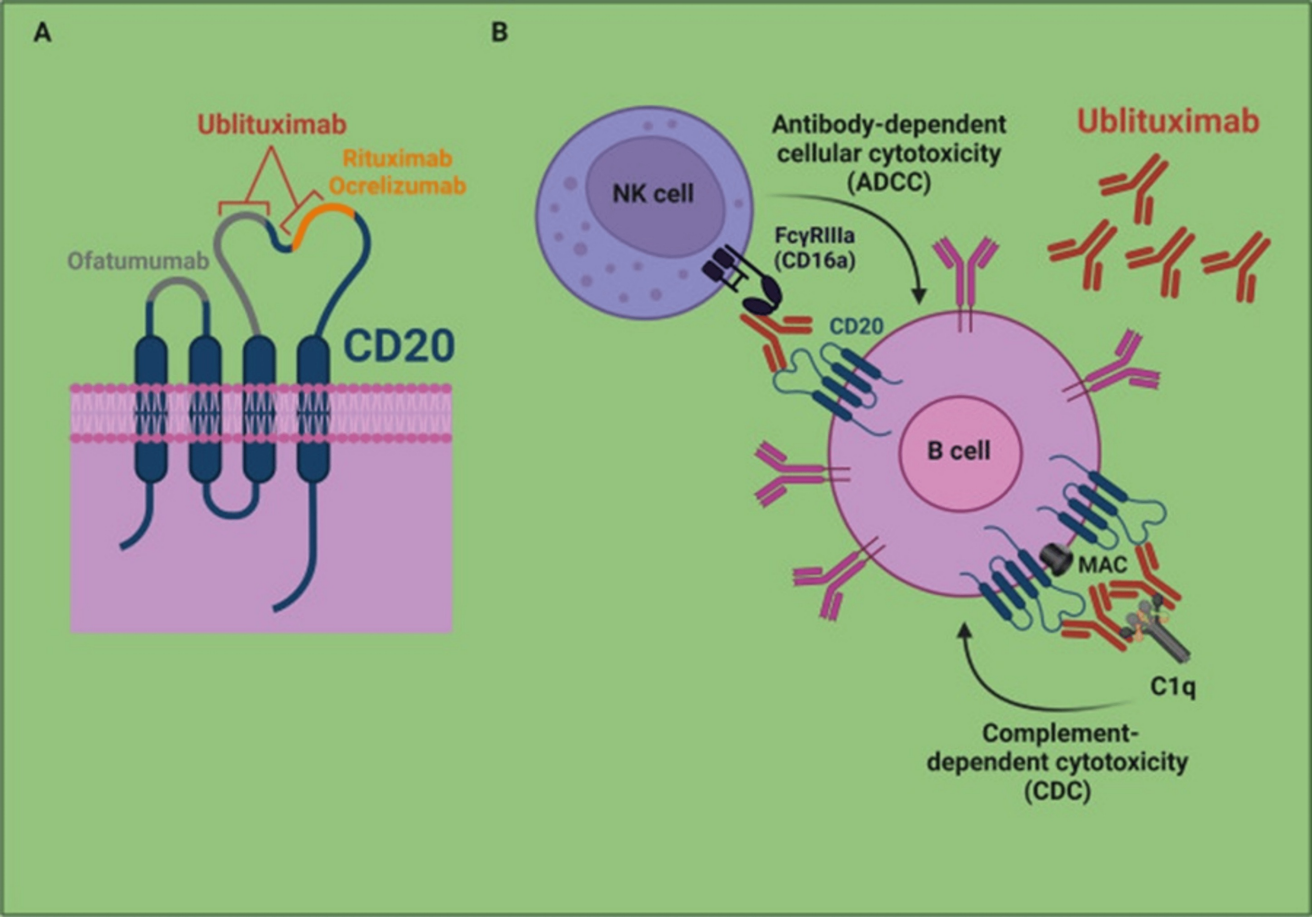
Mechanism of action of ublitixumab Image credit: Created by the Dr. Sai Vineeth Chitturi.

Ublituximab is a glycoengineered monoclonal antibody that selectively targets the CD20 expressed on B lymphocytes. By binding to CD20, ublituximab mediates B-cell depletion through antibody-dependent cellular cytotoxicity (ADCC) and complement-dependent cytotoxicity (CDC). The glycoengineering process enhances its affinity for FcγRIIIa receptors on immune effector cells, increasing ADCC activity compared to conventional anti-CD20 antibodies. B-cell depletion reduces inflammation and immune-mediated damage in MS, thereby decreasing disease activity and relapse rates [9]. Ublituximab and its site of action are presented in Figure 1.

Mechanism of action of natalizumab

**Figure 2:**
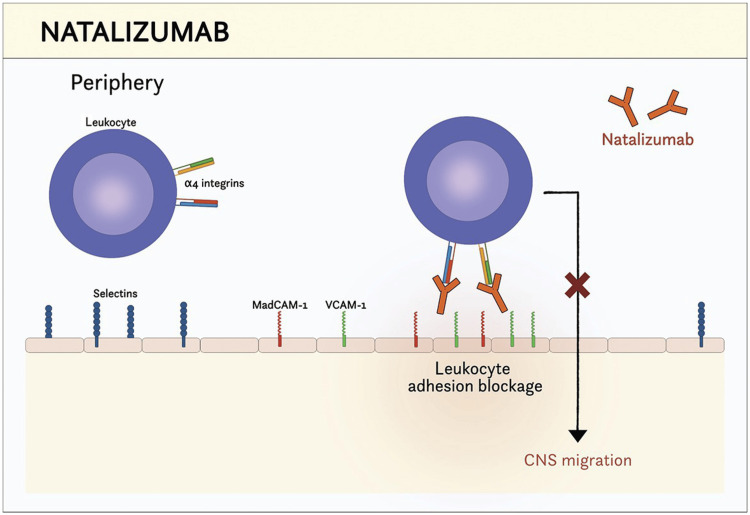
Mechanism of Action of Natalizumab Image credit: Created by the Dr. Sai Vineeth Chitturi.

Natalizumab is a monoclonal antibody that inhibits immune cell migration into the CNS by targeting the α4-integrin subunit of α4β1 and α4β7 integrins. These integrins play a crucial role in leukocyte adhesion to vascular cell adhesion molecule-1 (VCAM-1) on endothelial cells. By blocking this interaction, natalizumab prevents autoreactive lymphocytes from crossing the BBB and attacking myelin in the CNS. This mechanism significantly reduces inflammation, lesion formation, and relapse rates in MS patients [10]. Natalizumab and its site of action are presented in Figure 2.

Efficacy of ublituximab

The efficacy of ublituximab was evaluated in two phase III clinical trials, Ublituximab in Multiple Sclerosis Treatment Investigating Efficacy (ULTIMATE) I and II. These studies found that ublituximab significantly reduced annualized relapse rates (ARR) compared to teriflunomide. Specifically, ARRs for ublituximab were 0.08 and 0.09 in ULTIMATE I and II, respectively, whereas teriflunomide had higher ARRs of 0.19 and 0.18 [9]. Ublituximab shows encouraging efficacy, with majority of all ublituximab-treated patients demonstrating no clinical or MRI disease activity over the 11-month therapy period. New T2 lesions found on-study occurred predominantly before week 24, when the medicine might not have had its full impact [11]. MRI scans further confirmed reduced disease activity, showing a decrease in gadolinium-enhancing lesions.

A meta-analysis comparing ublituximab with other monoclonal antibodies, including natalizumab, found no significant difference in ARR reduction. The rate ratio for ARR between ublituximab and natalizumab was approximately 0.99 (95% CI: 0.59-1.65). Similarly, there were no notable differences in six-month confirmed disability progression (CDP), with a hazard ratio of 1.13 (95% CI 0.53-2.40) [12].

Infusion-related reactions (IRRs) were observed in approximately 43% of participants in the ULTIMATE I trial and about 50% in ULTIMATE II, both of which were phase III studies assessing ublituximab in individuals with RRMS. These reactions were mostly mild to moderate and tended to decrease with subsequent dosing. Infection rates were documented in 48% of subjects in ULTIMATE I and 61% in ULTIMATE II, with respiratory tract infections being the most common, generally presenting with mild or moderate severity. Over time, there was an increase in the proportion of patients showing immunoglobulin M (IgM) levels below the lower limit of normal, rising from about 1% at baseline to 13% at 48 weeks and 21% at 96 weeks. In contrast, IgG levels remained relatively stable, with around 6% of patients falling below the lower limit of normal throughout the study period [13].

Efficacy of natalizumab

The pathophysiology of acute inflammatory lesions in MS patients is driven by α4 integrin and the immune cells that express it. The mechanism involving the antagonism of α4integrin-dependent adhesion of leukocytes that move to areas of inflammation is compatible with the increase in circulating lymphocytes in patients treated with natalizumab. Both natalizumab dosages were linked to near-saturation of the α4β1 integrin receptor, and binding of natalizumab to α4β1 integrin may also prevent T lymphocyte activation or eradicate autoreactive T cells. After one month of treatment, there was a noticeable decrease in the quantity of new enhancing lesions, indicating that natalizumab prevents the onset of lesions. In summary, natalizumab had beneficial effects on clinical and imaging outcomes in patients with relapsing MS. Therapy was well tolerated during the six-month trial [10].

The AFFIRM (Natalizumab Safety and Efficacy in Relapsing Multiple Sclerosis) clinical trial demonstrated that natalizumab lowered the risk of sustained disability progression by 42% and reduced ARR by 68% over two years compared to placebo. MRI evaluations revealed a marked decline in new or enlarging T2-hyperintense lesions and gadolinium-enhancing lesions, indicating effective disease control [14]. Over a two-year period, natalizumab reduced the mean number of Gd+ lesions by 92% and the mean number of new or expanding T2-hyperintense lesions by 83% (both p<0.001). Another Phase 3 trial found that natalizumab with interferon (IFN) β-1a decreased the probability of persistent disability advancement by 24% (HR 0.76; 95% CI: 0.61-0.96; p=0.02) and the mean ARR by 55% at two years when compared to IFNβ-1a alone (p<0.001). Six percent of patients experienced a loss of efficacy due to persisting anti-natalizumab antibodies. Progressive multifocal leukoencephalopathy (PML) has an estimated 1:1000 chance of developing over the course of 18 months; the long-term risk for PML is unclear. The advantages and disadvantages of natalizumab support its use as a monotherapy for patients with quickly progressing severe RRMS and for RRMS with significant disease activity in spite of IFNβ treatment [15].

Treatment with natalizumab resulted in an 83% reduction in the number of new T2-hyperintense lesions over a two-year period compared to placebo (mean 1.9±9.2 lesions vs. 11.0±15.7 lesions; P<0.001). Additionally, natalizumab reduced the mean number of gadolinium-enhancing lesions by 92% at both one and two years. There was also a 76% reduction (P<0.001) in the mean number of new T1-hypointense lesions over two years, with an average of 1.1 lesions in the natalizumab group compared to 4.6 lesions in the placebo group. Notably, the mean reduction in brain parenchymal fraction (BPF), an indicator of brain atrophy, was comparable between the two groups [16].

Long-term observational studies, such as the Tysabri Observational Program (TOP), support natalizumab’s effectiveness. In this study, 23.9% of patients achieved confirmed disability improvement (CDI), with 51.8% experiencing CDI within the first year of treatment. Early initiation of natalizumab was linked to better long-term disability outcomes [17].

Comparative efficacy of ublituximab versus natalizumab

Although direct head-to-head studies between ublituximab and natalizumab are lacking, indirect comparisons suggest they offer similar benefits in controlling disease activity in RRMS. Treatment decisions should be based on individual patient characteristics, prior response to therapies, and potential safety risks.

Safety considerations

Each therapy presents unique safety concerns. Ublituximab was associated with a higher incidence of infections, including severe infections. Infusion-related reactions were reported in approximately 47.7% of patients receiving ublituximab. Natalizumab carries a risk of progressive multifocal leukoencephalopathy (PML), a severe brain infection. The therapeutic considerations for MS based on patient risk factors and the corresponding drug preferences are outlined in Table 1.

**Table 1:** Therapeutic Considerations for Multiple Sclerosis: Drug Preferences Based on Patient Risk Factors JC virus: John Cunningham virus; MS: multiple sclerosis; PML: progressive multifocal leukoencephalopathy.

Patient Factor	Preferred	Avoid
JC virus positive	Ublituximab	Natalizumab (PML risk)
Highly active MS	Natalizumab	None
History of severe infections	Natalizumab	Ublituximab
Pregnancy	Natalizumab (caution)	Ublituximab
Autoimmune conditions	Natalizumab	Ublituximab (caution)

## Conclusions

Ublituximab and natalizumab are both highly effective in managing RRMS, significantly reducing relapse rates and disease activity. For patients with RRMS, natalizumab continues to be a crucial component of the therapeutic regimen. It is very effective in preventing new MRI activity and lowering clinical relapses. But ublituximab's lower risk of PML and longer dosing interval may make it the safer and more convenient option for most patients. Future research focuses on creating new biomarkers that could enhance natalizumab's safety and more accurately predict a person's risk of PML. While direct comparative trials are unavailable, available data suggest they provide similar benefits. The selection of an optimal treatment should be individualized.
